# On Vaccine Inoculation

**Published:** 1802-01

**Authors:** 


### *To the Editors of the Medical and Phyfical Journal.*

GENTLEMEN,

THE fuccefs of Vaccine Inoculation has been commenfurate with its progrefs; thofe who doubted, in the infancy of its introduction, availed themfelves of actual experiment, and became, at an early period, true believers. Its ftrides were fo rapid, that the jargon of ignorance has been long fince filenced, and the promifed cafes which were to invalidate its fecurity, collected by profeffional jealoufy or perfonal animofity, are wifely withheld. It has the experimental fanction of all claffes of fociety in our own country, the public teftimony of our Navy and Army, and is becoming or become general in every part of the civilized world; and although to attempt a calculation of the immenfe number of lives it muft be the caufe of faving, to point out the horrid features of the loathfome difeafe it fupplants; in fhort, to enumerate all or any of its advantages to a Britifh Senator, would be only a repetition of what he has often heard, of that he is thoroughly convinced, yet has the Senate not voted its Difcoverer a remuneration. The object attained muft be of fufficient confequence to demand the gratitude of the nation; whilft foreign focieties are ftriving, therefore, which fhall firft beftow honours on our countryman, let us no longer hefitate to vote him more folid benefits. But fhould the legiflative body of this country ftill require more pointed teftimonies, in favor of this new, mild, and efficacicioufly preventive difeafe, previous to its liberally renumerating

 the the Difcoverer, the fuggeftion of your Correfpondent, J. H. in your laft number, certainly deferves attention, as the univerfal confent and adoption of medical men muft be regarded as irrefragable evidence. To further this plan, I fhould propofe that a Committee be formed in London, who fhall firft announce their object, and receive fubfcriptions to defray the expences of advertifements, poftage, &c. A fufficient fund being thus obtained, the fulleft and moft unequivocally worded teftimony in its favour fhould be drawn up, and publifhed a certain number of times in every London paper, requefting the written approbation of thofe medical men, from every part of this ifland, whofe experience warrants their conviction to the full extent of the circulated paper; and with the addition of thefe names, the Committee fhould lay the ftatement before the Chancellor of the Exchequer, praying that the perfon to whom the whole world is fo much indebted, might be amply rewarded by his own country.

It has been ftated, as an objection to remuneration, that Dr. Jenner had ample field to make a large fortune by the difcovery, which he neglected, whilft others were reaping the advantage. This objection furnifhes an argument againft itfelf, for it proves that the Doctor was fpending that time in informing others, which he might, on a narrower fcale of acting, have ufed to his own private emolument; and it is the fact, that the inftant his experiments had convinced his own mind, he fent the difcovery to the world. This whole time, then, became occupied in removing the doubts, and in combating the prejudices of his brother practitioners. His rooms were crouded with interrogating vifitors, and his hands were filled with letters, which left him little opportunity of attending to his own private aggrandifement. Dr. Jenner has acted as a man of fcience; as a man, who, feeling warmly for the interefts of humanity, preferred its fafety and its comfort to any felfifh motives: Such a conduct, it is feared, is too rare, but it cannot be too well rewarded.

*Wotton-under-Edge, Nov.* 19, 1801. D. T.

